# Pre‐Pandemic Prevalence of Post COVID‐19 Condition Symptoms in Adolescents

**DOI:** 10.1111/apa.70123

**Published:** 2025-06-06

**Authors:** Jesse Linton, Joshua Carmichael, Fiona Newlands, Amal Puri, Lana Fox‐Smith, Snehal M. Pinto Pereira, Anna Coughtrey, Roz Shafran, Terence Stephenson

**Affiliations:** ^1^ UCL Great Ormond Street Institute of Child Health London UK; ^2^ Division of Surgery & Interventional Science, Faculty of Medical Sciences University College London London UK

**Keywords:** adolescents, long COVID, post COVID condition, post COVID‐19 condition

## Abstract

**Aim:**

The emergence of post COVID‐19 condition (PCC) within adolescents has been characterised by a wide range of symptoms, raising concerns for young people's health and quality of life. However, many symptoms are non‐specific and there is considerable variation in symptom reporting. It is essential to understand how rates of these symptoms compare to the pre‐pandemic health of adolescents.

**Methods:**

A systematic search of academic literature and websites, using traditional and automated search systems, was undertaken to identify symptoms described in adolescents aged 10–19 years in the 30 years up to and including 2019. Studies were reviewed and symptom prevalence data extracted.

**Results:**

Twenty‐five sources (*n* = 483 097 participants) met the inclusion criteria, including longitudinal and cross‐sectional study designs. The description and prevalence of symptoms varied widely, but there was a high pre‐pandemic median prevalence of cough (13.6%), headache (30.0%), and fatigue (20.5%). These high prevalences highlight a gap in understanding of pre‐pandemic adolescent health and the need for comprehensive, serial symptom profiling.

**Conclusion:**

These findings provide a baseline of adolescent symptomatology prior to the emergence of PCC and provide important context for interpreting ongoing COVID symptoms. Data on PCC in adolescents should consider pre‐pandemic symptom prevalence.

**Abbreviations**
AP: Amal Puri
CloCk: Children & young people with Long COVID
CYP: CHildren and Young People
FN: Fiona Newlands
IQR: Interquartile Range
JC: Joshua Carmichael
JL: Jesse Linton
NIHR: National Institute for Health and Care Research
PCC: Post COVID Condition
PRISMA: Preferred Reporting Items for Systematic Reviews and Meta‐analysis
UKRI: UK Research and Innovation
WHO: World Health Organisation

**Summary**

- This study establishes baseline symptom prevalence in adolescents prior to the COVID‐19 pandemic, highlighting high pre‐pandemic rates of cough (13.6%), headache (30.0%), and fatigue (20.5%).
- The findings emphasise the need for serial symptom profiling to better understand adolescent health and differentiate post COVID‐19 condition symptoms from pre‐existing patterns.
- By providing a comprehensive review of pre‐pandemic symptomatology, this study offers essential context for interpreting ongoing COVID‐related symptoms in adolescents.

## 1 Research in Context

### 1.1 Evidence Before This Study

To identify, characterise and understand symptoms of PCC in young people, researchers and clinicians need a comprehensive picture of the physical and psychological symptoms that adolescents were describing prior to the pandemic. Initial scoping searches of these ‘historical control’ data were conducted on databases including PubMed using the following key words: “Large scale” AND “Longitudinal health” AND “study OR survey OR cohort” AND “paediatric OR pediatric” OR “children and young people” OR “CYP” OR “adolescent”. These searches highlighted that whilst several high‐quality studies exist that report the prevalence of mental and physical health symptoms in young people, few papers reported on more than three symptoms at a time.

### 1.2 Added Value of This Study

We reviewed studies of symptoms experienced by adolescents in the 30 years prior to the emergence of the COVID pandemic. Our findings highlight that the common symptoms implicated in PCC, such as headache, cough, fatigue, anxiety and depression, had high prevalences in adolescents prior to the pandemic. However, studies were variable and there are gaps in our understanding of pre‐pandemic adolescent health.

### 1.3 Implications of All the Available Evidence

Data reporting on the nature and prevalence of PCC in adolescents should consider the pre‐pandemic prevalence of many symptoms and should interpret findings within this context. There is a need for further comprehensive symptom profiling to ensure that future studies have a thorough and complete picture of baseline adolescent health.

## 2 Introduction

The emergence of Post COVID Condition (PCC) or “Long COVID” within adolescents has been characterised by a wide range of symptoms, including headaches, shortness of breath, fatigue and cough [1]. In efforts to define and standardise the clinical approach to diagnosing young people with PCC, multiple studies have investigated the phenotype, prevalence, risk factors and lasting effects of PCC within this population [1, 2]. This research has led to the following consensus definition for PCC in young people: ‘post COVID‐19 condition occurs in children and young people with a history of confirmed SARS‐CoV‐2 infection, with one or more persisting physical symptoms for at least 12 weeks after testing that cannot be explained by an alternative diagnosis. The symptoms may have an impact on everyday functioning, may continue or develop after SARS‐CoV‐2 infection, and may fluctuate over time’ [3] This research definition for adolescents with PCC largely aligns with the World Health Organisation clinical definition for Children and Young People [4].

The prevalence of PCC in children and young people varies depending on definition, study design, and inclusion of SARS‐CoV‐2 variants [5, 6, 7, 8, 9, 10], PCC can have considerable consequences for individuals, populations and economies [11], and understanding the impact of PCC is likely to be particularly important for adolescents given the potential long‐lasting consequences on psychological, social, and educational functioning in this population [12, 13, 14]. The most common persistent symptoms identified in adolescents after SARS‐CoV‐2 infection have been tiredness, headache, and shortness of breath [15]. The prevalence associated with each of these, as well as respiratory, gastrointestinal, psychological and other symptoms has been documented and varies in range and severity. With over 200 symptoms associated with PCC, there has been great difficulty in determining if these are direct sequelae of COVID‐19 infection or more complexly associated with the wider effects of the pandemic and subsequent lockdown [12].

Much research has been conducted to compare the possible PCC symptoms within children and young people (CYP) testing positive for SARS‐CoV‐2 and control groups to establish the prevalence of these symptoms. However, the prevalence of these non‐specific symptoms within the 10–19‐year‐old population prior to the COVID‐19 pandemic and subsequent emergence of PCC is unclear [16]. Exploring and defining a baseline prevalence of these symptoms is necessary to contextualise PCC prevalence. A comparative approach is crucial for developing effective interventions and providing a contextual basis for symptomatology to help in developing diagnostic, referral, and management pathways for adolescents affected by PCC.

## 3 Methods

This systematic synthesis of symptom prevalence follows the Preferred Reporting Items for Systematic Reviews and Meta‐analysis (PRISMA) guidelines [17]. Our primary objective was to explore and synthesise the prevalence of pre‐pandemic symptoms in adolescents to allow a comparison with those described by adolescents experiencing post COVID‐19 conditions. Given this specific focus, a systematic search and synthesis allowed us to efficiently gather relevant data and present a focused and detailed analysis of the specific symptoms associated with PCC.

### 3.1 Inclusion and Exclusion Criteria

We included studies which used data derived from surveys that directly asked CYP, and in some cases their parents and carers, about a range of symptoms described for PCC in published literature. Sources reflected general population samples. Sick or hospitalised populations were excluded. The listed symptoms for inclusion encompassed anxiety, bleeding, cough, diarrhoea, weight loss, constipation, joint pain/swelling, hair loss, depression, tremor/shakiness, problems swallowing, psychiatric symptoms, headache, fatigue, infectious symptoms, inflammatory conditions, sadness, obstructive sleep apnoea, body weight changes, pulmonary embolism, learning difficulties, hallucinations, deconditioning, pain, enuresis, decreased physical resilience, chronic respiratory failure, decreased motivation, nervousness, pulmonary symptoms, endocrine disorders and general wellbeing. The set of symptoms used in this paper were defined a priori from a meta‐analysis and systematic review that outlined 200 symptoms associated with post COVID‐19 condition.

The full symptom list was initially refined using the categories described in the Benhood et al. [1] systematic review. This was further adapted to produce a final list of 33 symptoms by splitting some broader categories (e.g., “weight changes” into “weight loss” and “weight gain”) and adding clinically relevant groups not explicitly included in the original review (e.g., “infectious symptoms”). The resulting list aimed to balance clinical breadth with feasibility for analysis.

The target population comprised adolescents aged 10–19 years old, according to the World Health Organisation's definition of adolescent [4]. Sources published between 1989 and 2019 were included in this synthesis. Although the DEVONagent Pro [18] search strategy specified English language sources, some primary sources were identified in other languages. Translation tools, such as Microsoft Translator, were used where necessary. Efforts were made to prioritise data from primary sources and therefore, studies were excluded if they reported secondary analyses of primary data sources or included repeated datasets. Papers were also excluded if they did not report prevalence data, were prior to 1989 or after 2019, or if data were inaccessible.

### 3.2 Systematic Search Strategy

Sources were identified in two ways.

First, a systematic search of the academic literature was conducted using PubMed on 29th April 2024. Search terms were:

“(Large scale) AND ((Longitudinal health) AND (study OR survey OR cohort)) AND ((paediatric OR pediatric) OR (children and young people) OR (CYP) OR (adolescent))”.

Second, a systematic search of websites was conducted on 25th January 2024 using DEVONagent Pro [18], a software package that searches multiple online sources and provided a summary of results based on the search terms:

“Language: English; “Ignore Diactrics” checked; “Fuzzy” checked (allow alternative spelling); Similar pages NOT filtered; Archived pages NOT filtered. Search terms in Title, Text, URL, Keywords, Description; HTML & XHTML pages, Atom, RSS & JSON feeds, Plain text documents, PDFs, and Microsoft word documents were searched; Searches across Plugins: Bing, Google, Yahoo!; Maximum results limit per Plugin: 1000. Search terms were like those used for PubMed: “Large scale” AND “Longitudinal health” AND “study OR survey OR cohort” AND “paediatric OR pediatric” OR “children and young people” OR “CYP” OR “adolescent””.

Citations were also reviewed to identify primary sources for data extraction.

### 3.3 Study Selection and Data Extraction

Manual review of each source was performed on results from PubMed and DEVONagent Pro [18] via reference to identical inclusion and exclusion criteria. JC screened the academic literature; FN and JL independently screened each automated search source, with no automated tools utilised. Sources identified from the search where the original paper was in non‐English languages were not excluded, and translation tools were used where necessary. Sources were excluded without screening if duplicated or irrelevant by title alone.

### 3.4 Data Collection

Data from included papers were extracted into Microsoft Excel by JL, AP, and JC. The main aim was to extract prevalence data provided. Study design and population characteristics (e.g., gender) were also collated (Table S1). Unclear data were clarified through secondary sources when possible. For example, we reviewed secondary related websites or articles to find information (e.g., number or age of participants). Data that required special access was sought when possible. Unavailable data have been noted in the results. Results were synthesised narratively given the high heterogeneity of included studies.

### 3.5 Outcomes and Variables

Prevalence data were sought for the listed symptoms. Data metrics, for example, confidence intervals and standard errors, were inconsistent between studies; however, where available, this data has been shown (Tables S2 and S3). Data on the age of participants and year of data collection were collated. Both adolescent self‐reports and parent/caregiver reports were included. Three disputes over symptom definition or categorization (psychiatric symptoms, endocrine disorders, and infectious symptoms) were resolved through consensus. Other variables recorded included question characteristics (e.g., subjective characterisation of general health) and variation of symptom definition.

### 3.6 Effect Measures

Prevalence data are presented as percentages (Tables S2–, S4). Where outcomes were presented as multiple discrete variables, averages were calculated and reported (Table 1). Thresholds for high prevalence were defined by the prevalence rate exceeding 10%. Low prevalence thresholds were defined by the prevalence rate lower than 2%.

**TABLE 1:** Symptom frequency and prevalence (%) with median and IQR.

Symptom	Frequency of symptom prevalence	Prevalence (%) median (IQR)
Anxiety	21	19·90 (6.60, 31.20)
Body weight changes	1	56·80
Chronic respiratory failure	6	10·65 (9.95, 27.80)
Constipation	3	7·19 (4.28, 7.32)
Cough	30	13·60 (9.52, 32.36)
Depression	29	21.20 (6.30, 27.50)
Diarrhoea	3	5·54 (3.39, 5.77)
Endocrine disorders	4	0·30 (0.19, 0.51)
Enuresis	2	3·35 (2.63, 4.08)
Fatigue	9	20.50 (14.89, 29.00)
General wellbeing	19	85.3 (84.05, 88.32)
Hallucinations	8	7·85 (2.85, 14.03)
Headache	33	30.00 (26.82, 56.40)
Infectious symptoms	2	18·10 (16.05, 20.15)
Inflammatory condition	4	3·45 (0.78, 6.70)
Joint pain or swelling	7	7·95 (1.86, 12.87)
Learning difficulties	4	8·45 (8.18, 8.53)
Nervousness	20	41.50 (34.65, 54.23)
Pain	37	20.00 (14, 32.50)
Problems swallowing	2	1·30 (1.20, 1.40)
Psychiatric problem	6	15·80 (13.55, 18.05)
Pulmonary symptoms	11	11·60 (9.00, 19.15)
Sadness	20	29·40 (21.6, 34.80)

*Note:* Displays the frequency, prevalence (%) with median and interquartile range for each symptom extracted from the papers. Symptoms without associated prevalence data have been excluded from the table.

## 4 Results

**FIGURE 1:**
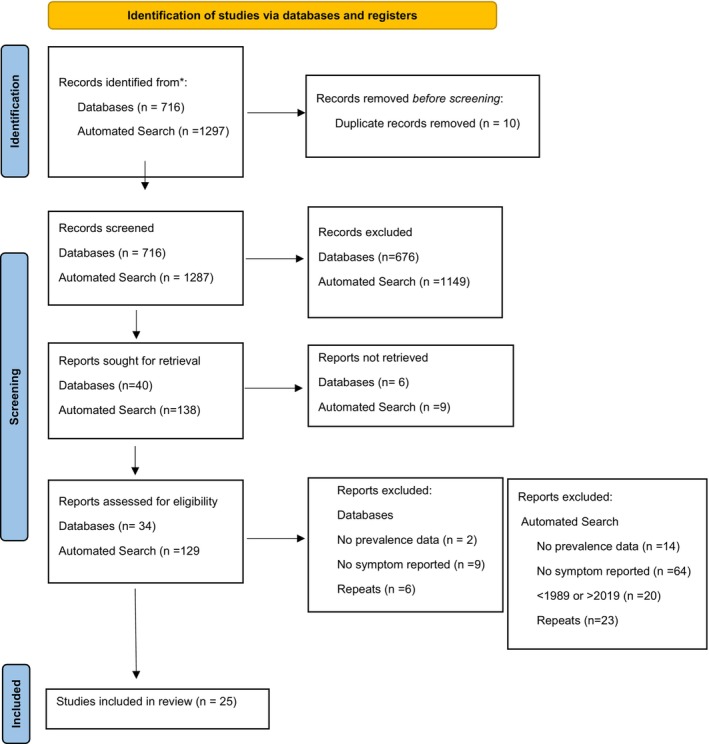
PRISMA flow diagram of included studies. The Figure shows the flow of study identification and selection. The original database search resulted in 716 records from PUBMED. The original Automated search, via DEVONpro Agent, resulted in 1297 records from websites. After duplicates were removed, there were 716 unique reports eligible for title and abstract screening from databases and 1287 from the automated search. The first phase of screening excluded 676 reports from databases and 1149 from the automated search. Following this, 163 reports were assessed for eligibility through screening of full‐text articles. The second phase of screening excluded 117 reports from the automated search for the following reasons: 64 reported on no relevant PCC symptoms, 14 had no prevalence data for PCC symptoms discussed, 20 were prior to 1989 or later than 2019, and 23 were secondary sources. From the databases, 17 reports were excluded for the following reasons: 9 reported on no relevant PCC symptoms, 2 had no prevalence data for PCC symptoms discussed and 6 were secondary sources. Twenty‐five studies were included in the review. *n*, number of studies. PRISMA, preferred reporting items for systematic reviews and meta‐analyses.

Our systematic search findings are summarised in the PRISMA flow diagram (Figure 1). Initial searches identified 716 (database) and 1297 (automated search) articles. Following the removal of duplicates (*n* = 10) and articles excluded during initial title screening from the automated search (*n* = 676) and databases (*n* = 1149), 178 were reviewed against the inclusion and exclusion criteria. 15 sources were irretrievable. After the exclusion of 138 studies, 25 papers [19, 20, 21, 22, 23, 24, 25, 26, 27, 28, 29, 30, 31, 32, 33, 34, 35, 36, 37, 38, 39, 40, 41, 42, 43] were included in this systematic symptom synthesis.

### 4.1 Study Characteristics

Of the 25 studies that met the inclusion criteria, 18 were longitudinal studies [19, 20, 21, 22, 23, 24, 25, 26, 27, 28, 29, 30, 31, 32, 33, 42, 43] and 7 were cross‐sectional studies [34, 35, 36, 37, 38, 39, 40, 41]. In total, the datasets comprised of 483 097 participants. The median number of participants was 6054, with the largest study including 120 115 and the smallest, 431 adolescents. Studies were conducted in various regions across the United Kingdom and internationally, including the United States [24, 25, 27, 32, 34, 39], China [35, 41, 42] and Finland [28]. While studies were included if information could be extracted for ages 10–19 inclusive, there was variation in the specific age groups represented within each study. Ages 10, 18 and 19 were the least represented, while ages 12–15 years were the most frequently included. Cohorts were often divided into younger (11–15‐year‐olds) and older adolescents (16–19‐year‐olds) (Table S1). Setting also differed between studies, but most often, adolescents were surveyed for data collection within school or in dedicated centres. Demographic data varied, although sex differences were most consistently presented within studies (Table S1). This is represented by the addition of subsets for studies where this data was available.

Within the included datasets, prevalence data varied significantly according to symptom definition, methodology, and population characteristics (Table S1). Seven core symptoms were frequently represented, appearing in more than 20 study subsets. These symptoms are highlighted as “high prevalence” symptoms (Table S2). In contrast, other gastrointestinal, musculoskeletal and other non‐specific symptoms (Table S3) had data available between 1 and 19 times, indicating less consistency in reporting. Notably, no prevalence data was extracted for bleeding, weight loss or gain, hair loss, tremor, obstructive sleep apnoea, pulmonary embolism, deconditioning, or decreased physical resilience (Table 1). The number of symptoms reported per study also varied, with a median of three symptoms per study. The Avon Longitudinal Study of Children and Parents: Teenage Focus Surveys provided the most comprehensive data, with 11 symptoms which feature commonly in PCC reporting [22]. The prevalence of the commonest symptoms across studies can be seen in Figures S2–S6.

There were major differences in methodology between studies. Time frames for symptom recall differed in specificity with some using broad categories as in the National Survey of Children's Health [34], where participants were asked about their health “in the past 12 months”, compared to, for example, adolescents asked to recall if they had experienced cough “at the time of the survey” in the Young‐Hunt study [26]. These differences complicate direct comparisons since prevalences vary according to question design. These contrasting methodologies are presented where applicable (Table S1).

### 4.2 High Prevalence Symptoms

The median prevalence and interquartile range of symptoms has been represented (Table 1). Several non‐specific symptoms including headache (30.0%), cough (13∙6%), fatigue (21∙5%) and pain (20∙0%) showed prevalence rates above 10%. The prevalence of chronic respiratory issues (10∙65%) highlights pre‐pandemic concerns associated with conditions including asthma, allergies, and environmental exposures. However, there is also considerable variability between studies for each given symptom, with interquartile ranges of headache (26.82, 56.40), cough (9∙52, 32∙36) and fatigue (14∙89, 29∙00). Anxiety (19∙9%) and depression (20∙5%) were also high in young people (Table 1). Despite the prevalence of specific symptoms, 85% (IQR = 84∙05, 88∙32) of adolescents reported general well‐being across studies, suggesting a perceived baseline of good health. Gastrointestinal symptoms like diarrhoea (4·26%), constipation (5·43%), and problems swallowing (1·3%) each appeared in only one study (Table S5–, S7).

## 5 Discussion

The overall aim of this systematic synthesis was to collate existing pre‐pandemic symptom prevalence data to establish a baseline frequency for the symptoms subsequently found to be associated with PCC in adolescents. Such an analysis would enable the differentiation of symptoms that exhibited high prevalence prior to the emergence of PCC and those whose prevalence could more be isolated as specific to PCC.

Overall, there was overlap in symptomatology between PCC and other commonly occurring chronic and acute conditions experienced by this age group. Our review demonstrated that fatigue, headache, cough, pain and psychological distress were common health concerns before the pandemic (Tables S2 and S4). Further, our findings underscored the gap in existing data, the importance of comprehensive health assessment and the establishment of an all‐encompassing baseline reference for adolescent health. The gap in knowledge was widened by the variability in methodological differences in study design, inconsistencies in symptom definition and differentiation, and diverse recall data in the research included in this review.

This highlights the need for standardised symptom definitions and methodological approaches in improving consistency for future studies. For example, “headache” was a prevalent symptom experienced by adolescents (Tables S3–, S6) but the specific definition of “headache” varied between subsets.

Furthermore, some studies were concerned with the adolescents' experience of headache within the last 2 weeks [28], others asked adolescents if they had *ever* experienced headache [25], and there were studies which did not qualify a length of time, opting for a qualitative approach in the spectrum of adolescents' experience of this symptom [32, 38]. This latter approach led to higher prevalence as responses of “sometimes” and “often” were averaged. The wide range and individualised experience of headache between cohorts are noted to have a potential influence on reported prevalence and may partly explain the data we found.

The diversity of survey design between studies was often mirrored for data collected for cough, joint pain, depression, anxiety, fatigue, headache, sadness, hallucination and pain.

### 5.1 Implications

High rates of cough, fatigue, headache, and pain have been described in studies of adolescents with PCC (Tables S2 and S4). A major challenge has been whether the apparent high prevalence of these symptoms was also found in adolescents self‐reporting their health pre‐pandemic. Our findings indicate that this was the case for many symptoms. Our study underscores the need for comprehensive, robust health baseline data to avoid over‐attribution of symptoms because of the pandemic. This study also highlights the importance of ongoing monitoring of persistent symptoms and collation of symptom prevalence that is accessible to researchers tasked with profiling emerging conditions where there is clinical uncertainty.

A second major challenge has been to determine whether these symptoms solely stem from the effects of previous SARS‐CoV‐2 infection or overlap with other conditions affecting adolescents or are the consequences of the social distancing measures and societal impact of a global pandemic. This study cannot answer that question. It can only provide a baseline comparison of symptoms in adolescents who have lived through a pandemic compared to pre‐pandemic normative data.

### 5.2 Limitations

The systematic synthesis process and the limitations within included studies presented challenges affecting the reliability of results. Despite efforts to use data representative of the period from the 30 years prior to the pandemic, there was a scarcity of studies from certain periods, leading to potential gaps within the data. This may, in part, be due to secular trends associated with acceptability of and subsequent increased reporting in symptoms, especially in mental health reporting. The lack of prevalence data for some symptoms may indicate limitations in the methods of data acquisition and screening used. The variability between number, type and method of symptom prevalence data within studies underscored the inconsistencies of data collation. One study (National Survey of Children's Health) included headaches both under chronic pain and as a separate symptom. To avoid potential double‐counting, we treated these as distinct items while acknowledging the overlap in symptom classification. Further, the defining of a threshold for “high prevalence” symptoms was arbitrarily set at 10%. Future research is needed to address these gaps and inconsistencies to develop a more comprehensive understanding of PCC in adolescents. The search for large‐scale, longitudinal cohort studies also meant that some potentially relevant information sources were not included. Some data were omitted due to studies requiring lengthy application processes, archived or held behind paywalls, such as those involving the Hospital Episode Statistics. These limitations may impact the reliability of results.

## 6 Conclusion

Our synthesis revealed that many symptoms associated with PCC were prevalent among adolescents before the pandemic, suggesting a complex interplay between pre‐existing symptomology and new symptoms induced by COVID‐19. Researchers and healthcare providers should consider such baseline data when diagnosing PCC to avoid misattributing long‐standing health issues or common episodic adolescent symptoms to the aftermath of the virus. The collection of prevalence data to establish baseline rates of adolescent health is an important area for future research to enable the contextualisation of emerging health conditions.

## Author Contributions

**Jesse Linton:** conceptualization, methodology, data curation, formal analysis, writing – original draft, writing – review and editing. **Joshua Carmichael:** conceptualization, methodology, data curation, formal analysis, writing – review and editing. **Fiona Newlands:** methodology, data curation, resources, writing – review and editing. **Amal Puri:** methodology, data curation, writing – review and editing. **Lana Fox‐Smith:** conceptualization, resources, project administration, writing – review and editing. **Snehal M. Pinto Pereira:** methodology, supervision, writing – review and editing. **Anna Coughtrey:** conceptualization, supervision, writing – review and editing, writing – original draft. **Roz Shafran:** conceptualization, methodology, supervision, resources, writing – review and editing. **Terence Stephenson:** conceptualization, methodology, supervision, writing – review and editing.

## Disclosure

All research at Great Ormond Street Hospital NHS Foundation Trust and UCL Great Ormond Street Institute of Child Health is made possible by the NIHR Great Ormond Street Hospital Biomedical Research Centre. The views expressed are those of the authors and not necessarily those of the NHS, the NIHR, UKRI or the Department of Health and Social Care.

## Conflicts of Interest

T.S. is Chair of the Health Research Authority.

## Supporting information


Table S1.

Table S2.

Table S3.

Table S4.

Table S5.

Table S6.

Table S7.
