# Supplementary material for: Pre‐Pandemic Prevalence of Post COVID‐19 Condition Symptoms in Adolescents
Source: Acta Paediatr. 2025 Jun 6;114(9):2116–23. doi: 10.1111/apa.70123 (PMC12336932; doi:10.1111/apa.70123)
Supplement: Supplementary file 1 — Table S1. [file APA-114-2116-s006.docx]

| **Table S1: Study Characteristics** | | | | | |
| --- | --- | --- | --- | --- | --- |
| **Study** | | **Study Type** | **Year of data collection** | **N** | **Age of Participants (Years)** |
| **Avon Longitudinal Study of Children and Parents** | |  |  |  |  |
| **Subset** | Teenage focus survey 1 | Cross-sectional | 2004 | 6832 | 12·5 |
|  | Teenage focus survey 2 |  | 2006 | 6141 | 13·5 |
|  | Teenage focus survey 3 |  | 2008 | 5509 | 15·5 |
| **Children's Health Study** | |  |  |  |  |
| **Subset** | Age 10 Cohort | Longitudinal | 1993 - 2012 | 4,602 | 10 |
|  | Age 15 Cohort |  | 1993 - 2012 | 2,338 | 15 |
| **Childrens Wellbeing Measures ONS** | |  |  |  |  |
| **Subset** | Child General happiness with health | Cross-sectional | 2012 | 3942 | 10 - 15 |
|  | Child General happiness with health |  | 2013 | 1504 | 10 - 15 |
|  | Child General happiness with health |  | 2014 | 3013 | 10 - 15 |
|  | Child General happiness with health |  | 2015 | 1565 | 10 - 15 |
| **China Nutrition and Health Surveillance of Children and Lactating Women** | | Cross-sectional | 2016-17 | 77,000 | 10 - 17 |
| **Hawaiian High Schools Health Survey** | | Cross-sectional | 2019 | 5,879 | 14 - 19 |
| **Health Behaviours of School age Children** | |  |  |  |  |
| **Subset** | 11-year-old boys |  | 2018 | 679 | 11 |
|  | 11-year-old girls | Longitudinal | 2018 | 628 | 11 |
|  | 13-year-old boys |  | 2018 | 691 | 13 |
|  | 13 year old girls |  | 2018 | 505 | 13 |
|  | 15-year-old boys |  | 2018 | 431 | 15 |
|  | 15-year-old girls |  | 2018 | 427 | 15 |
|  | Total (girls) |  | 2018 | 1561 | 11,13,15 |
|  | Total (boys) |  | 2018 | 1801 | 11,13,15 |
|  | Total (all) |  | 2018 | 10121 | 11,13,15 |
| **Finnish IEQ and Symptoms study** | |  |  |  |  |
| Subset | Grade 3-6 pupils |  | 2016-18 | 8775 | 10 - 13 |
|  | Grade 7-9 pupils | Longitudinal | 2016-18 | 3410 | 13 - 15 |
| **Leicestershire Respiratory Cohort** | |  |  |  |  |
| **Subset** | "coughing more" |  | 1998 | 8700 | 10 - 13 |
|  | "cough with cold" |  | 1998 | 8700 | 10 - 13 |
|  | "cough without cold" |  | 1998 | 8700 | 10 - 13 |
|  | night cough | Longitudinal | 1998 | 8700 | 10 - 13 |
|  | "coughing more" |  | 1998 | 8700 | 14 - 17 |
|  | "cough with cold" |  | 1998 | 8700 | 14 - 17 |
|  | "cough without cold" |  | 1998 | 8700 | 14 - 17 |
|  | night cough |  | 1998 | 8700 | 14 - 17 |
| **Mental health of children and Young People Surveys** | |  |  |  |  |
| **Subset** | Boys | Cross-sectional | 1999 | 2310 | 11 - 15 |
|  | Girls |  | 1999 | 2299 | 11 - 15 |
|  | Boys |  | 2004 | 1783 | 11 - 15 |
|  | Girls |  | 2004 | 1654 | 11 - 15 |
|  | All 11-16 |  | 2017 | 3121 | 11 - 16 |
|  | All 17-19 |  | 2017 | 936 | 17 - 19 |
| **Millenium cohort study - Age 14 sweep** | | Longitudinal | 2015 | 11,875 | 14 |
| **National Comorbidity Study-Adolescent Supplement** | | Longitudinal | 2001-04 | 10148 | 13 - 17 |
| **National Longitudinal Study of Adolescent Health** | | Longitudinal | 1996 | 14,738 | 11 - 18 |
| **National Longitudinal Study of Youth** | | Longitudinal | 1997 | 8984 | 12 - 16 |
| **Olympic Regeneration in East London (ORIEL)** | |  |  |  |  |
| **Subset** | Male | Longitudinal | 2012 | 3106 |  |
|  | Female |  | 2012 |  | 11 - 12 |
| **Online social networking addiction and depression** | | Longitudinal | 2014 | 4237 | 13-15 |
| **Prevalence of mental health problems in schools** | | Longitudinal | 2019 | 28,160 | 10- 19 |
| **Prevalence, severity and risk factors of asthma, rhinitis and eczema in a large group of Chinese schoolchildren** | | Longitudinal |  |  |  |
| **Subset:** Teenage cohort 13-18 years | |  | 2012-2013 | 2614 | 13 - 18 |
| **Project on Human Development in the Chicago Neighbourhoods** | |  |  |  |  |
| **Subset:** | Wave 1, cohort 12 | Longitudinal | 1994-97 | 820 | 10 - 13 |
| Youth Self Report | Wave 1, cohort 15 |  | 1994-97 | 696 | 13 - 16 |
|  | Wave 2, cohort 12 |  | 1997-00 | 670 | 12 - 17 |
|  | Wave 2, Cohort 15 |  | 1997-00 | 538 | 15 - 19 |
|  | Wave 3 cohort 9 |  | 2000-01 | 596 | 11 - 15 |
|  | Wave 3, cohort 12 |  | 2000-01 | 572 | 15 - 18 |
| **Subset:** Child Behaviour Checklist | Wave 1, cohort 12 |  | 1994-97 | 696 | 10 - 13 |
|  | Wave 1, cohort 15 |  | 1994-97 | 820 | 13 - 16 |
|  | Wave 2, cohort 12 |  | 1997-00 | 680 | 12 - 17 |
|  | Wave 2, Cohort 15 |  | 1997-00 | 549 | 15 -19 |
|  | Wave 3 cohort 9 |  | 2000-01 | 598 | 11 - 15 |
|  | Wave 3, cohort 12 |  | 2000-01 | 574 | 15 -18 |
| **Time trends in adolescent mental health** | |  |  |  |  |
| **Subset** | Boys | Longitudinal | 1999 | 2379 | 15 -16 |
|  | Girls |  | 1999 | 2199 | 15 -16 |
| **Tokyo TEEN Cohort** | |  |  |  |  |
| **Subset** | Wave 1 | Longitudinal | 2012-15 | 3171 | 10 - 16 |
|  | Wave 2 |  | 2014-17 | 3007 | 10 - 16 |
| **Tracking Adolescents Individual Lives Surveys** | |  |  | 2773 |  |
| **Subset** | Girls T1 | Cross-sectional | 2000-19 |  | 10 - 12 |
|  | Boys T1 |  | 2000-19 |  | 10 - 12 |
|  | Girls T2 |  | 2000-19 |  | 10 - 12 |
|  | Boys T2 |  | 2000-19 |  | 10 - 12 |
| **National Survey of Children’s Health** | |  |  |  |  |
| **Subset** | 2016 | Cross-sectional | 2016 | 20,292 | 12 - 17 |
|  | 2017-8 |  | 2017-18 | 21,359 | 12 - 17 |
|  | 2019 |  | 2019 | 12,171 | 12 - 17 |
| **What about Youth Survey** | | Cross-sectional | 2014 | 120,115 | 15 |
| **Young HUNT study** | |  |  |  |  |
| **Subset:** | Dry cough, without cold | Longitudinal | 1995 - 97 | 8983 | 13 - 19 |
| Young Hunt 1 | Cough>14 days |  | 1995 - 97 | 8983 | 13 - 19 |
|  | cough at time of survey |  | 1995 - 97 | 8983 | 13 - 19 |
|  | cough within 14 days |  | 1995 - 97 | 8983 | 13 - 19 |
|  | Other symptoms |  | 1995 - 97 | 8983 | 13 - 19 |
| **Subset:** | Dry cough, without cold |  | 1999 - 00 | 2399 | 13 - 19 |
| Young Hunt 2 | Cough>14 days |  | 1999 - 00 | 2399 | 13 - 19 |
|  | cough at time of survey |  | 1999 - 00 | 2399 | 13 - 19 |
|  | cough within 14 days |  | 1999 - 00 | 2399 | 13 - 19 |
|  | Other symptoms |  | 1999 - 00 | 2399 | 13 - 19 |
| **Subset:** | Dry cough, without cold |  | 2006 - 08 | 8200 | 13 - 19 |
| Young Hunt 3 | Cough>14 days |  | 2006 - 08 | 8200 | 13 - 19 |
|  | cough at time of survey |  | 2006 - 08 | 8200 | 13 - 19 |
|  | cough within 14 days |  | 2006 - 08 | 8200 | 13 - 19 |
|  | Other symptoms |  | 2006 - 08 | 8200 | 13 - 19 |
| **Understanding Society** | | Longitudinal | 2009 - 18 | 40,000 (households) | 10 - 15 |
