# Supplementary material for: Pre‐Pandemic Prevalence of Post COVID‐19 Condition Symptoms in Adolescents
Source: Acta Paediatr. 2025 Jun 6;114(9):2116–23. doi: 10.1111/apa.70123 (PMC12336932; doi:10.1111/apa.70123)
Supplement: Supplementary file 2 — Table S2. [file APA-114-2116-s004.docx]

**Supplementary Table 2: Prevalence by Study for High Prevalence Symptoms**

|  |  |  |  |  |  |  |  |  |  |
| --- | --- | --- | --- | --- | --- | --- | --- | --- | --- |
| **Study** | **Headache**  (%) | **Study** | **Pain**  (%) | **Study** | **Cough**  (%) | **Study** | **Pulmonary Symptoms** (%) | **Study** | **Fatigue**  (%) |
| Finnish IEQ and Symptoms study  Grade 3-6 pupils  Grade 7-9 pupils | 20·5  57·9 | Avon Longitudinal Study of Children and Parents  Teenage focus survey 1  Teenage focus survey 2 | 4·2    2·7 | Avon Longitudinal Study of Children and Parents  Teenage focus survey 3 | 5·2 | Avon Longitudinal Study of Children and Parents  Teenage focus survey 1 (current viral infection)  Teenage focus survey 2 (cold) | 14·2    11·6 | Avon Longitudinal Study of Children and Parents  Teenage focus survey 1  Teenage focus survey 2 | 5·3    7·2 |
| Hawaiian High Schools Health Survey | 17·2 | Health Behaviours of School age Children  11 year old boys  11 year old girls  13 year old boys  13  year old girls  15 year old boys  15 year old girls  Total (all) | 14  14  15  24  14  27  20 | Children's Health Study  Bronchitic symptoms Age 10 (4602)  Bronchitic symptoms Age 15 | 44·03    33·43 | Children's Health Study  Bronchitic symptoms Age 10 (4602)  Bronchitic symptoms Age 15 | 44·03    33·43 | Finnish IEQ and Symptoms study  Grade 3-6 pupils  Grade 7-9 pupils | 20·5  57·9 |
| Health Behaviours of School age Children  11 year old boys  11 year old girls  13 year old boys  13  year old girls  15 year old boys  15 year old girls  Total (all) | 19  25  25  41  29  49  30 | National Comorbidity Study-Adolescent Supplement | 0·548 | Finnish IEQ and Symptoms study  Grade 3-6 pupils  Grade 7-9 pupils | 2·4  9·4 | Finnish IEQ and Symptoms study  Grade 3-6 pupils  Grade 7-9 pupils | 21·2  17·1 | National Comorbidity Study-Adolescent Supplement | 14·89 |
| National Comorbidity Study-Adolescent Supplement | 26·82 | National Longitudinal Study of Adolescent Health  Chest pain  Stomach pain | 0·9  17·1 | Leicestershire Respiratory Cohort  "coughing more"  "cough with cold"  "cough without cold"  night cough  "coughing more"  "cough with cold"  "cough without cold"  night cough | 10  67  47  22  11  68  55  20 | National Longitudinal Study of Adolescent Health | 7·5 | Tracking Adolescents Individual Lives Surveys  Girls T1  Boys T1  Girls T2  Boys T2 | 20  22·5  43·1  29 |
| National Survey of Children’s Health (US population study)*  2016  2017-8  2019 | 5·8   (95% CI  [5.1 - 6.6])  5·6  (95% CI  [5.0 - 6.3])  5·5  (95% CI  [4.5 - 6.7]) | National Survey of Children’s Health (US population study)**  2016  2017-8  2019 | 11·7   (95% CI  [10.7 - 12.8])  12·2   (95% CI  [11.3 - 13.3])  13·7  (95% CI  [12.3 - 15.1]) | National Longitudinal Study of Adolescent Health | 2 | National Survey of Children’s Health (US population study)  2016  2017-8  2019 | 9·6   (95% CI  [8.6 - 10.6])  8·5  (95% CI  [7.6 - 9.5])  9·5   (95% CI  [8.3 - 10.9]) |  |  |
| Project on Human Development in the Chicago Neighbourhoods  PHDCN - Youth Self Report  Wave 1, cohort 12  Wave 1, cohort 15  Wave 2, cohort 12  Wave 2, cohort 15  Wave 3 cohort 9  Wave 3, cohort 12  PHDCN - Child Behaviour Checklist  Wave 1, cohort 12  PHSCN - Parent/carer reported symptoms  Wave 1, cohort 15  Wave 2, cohort 12  Wave 2, cohort 15  Wave 3 cohort 9  Wave 3, cohort 12 | 57·6  60·4  56·4  51·5  60·2  59·3      28·5      29·8  28·1  26·4  29·5  32·8 | Project on Human Development in the Chicago Neighbourhoods  PHDCN - Youth Self Report  Wave 1, cohort 12  Wave 1, cohort 15  Wave 2, cohort 12  Wave 2, cohort 15  Wave 3 cohort 9  Wave 3, cohort 12  PHDCN - Child Behaviour Checklist  Wave 1, cohort 12  PHSCN - Parent/carer reported symptoms  Wave 1, cohort 15  Wave 2, cohort 12  Wave 2, cohort 15  Wave 3 cohort 9  Wave 3, cohort 12 | 32·5  36·6  33  41·6  37·8  38·8      13·6      18·9  16·6  20  16·4  19·3 | National Survey of Children’s Health (US population study)  2016  2017-8  2019 | 9·6   (95% CI  [8.6 - 10.6])  8·5   (95% CI  [7.6 - 9.5])  9·5   (95% CI  [8.3 - 10.9]) | Young HUNT study  Young Hunt 1 other symptoms  Young Hunt 2 other symptoms | 3·11    3·42 |  |  |
| Tracking Adolescents Individual Lives Surveys  Girls T1  Boys T1  Girls T2  Boys T2 | 67  60·8  61·4  45·9 | Tracking Adolescents Individual Lives Surveys  Girls T1  Boys T1  Girls T2  Boys T2 | 43·1  39·1  28·5  23·1 | Young HUNT study  Young Hunt 1  “Dry cough, without cold”  “Cough>14 days”  “Cough at time of survey”  Cough within 14 days  Young Hunt 2  “Dry cough, without cold”  “Cough>14 days”  “Cough at time of survey”  “Cough within 14 days”  Young Hunt 3  “Dry cough, without cold”  “Cough>14 days “  “Cough at time of survey”  “Cough within 14 days “ | 13·58    1·82  18·2    29·13    13·12    2·49  13·6    23·46      12·41    18·18  36·92    35·68 |  |  |  |  |
| Understanding Society: Longitudinal Teaching Dataset, Waves 1-9, 2009-2018 | 38·8 | Young HUNT study 1  Young Hunt 1  Stomach Pain  Neck Pain  Young Hunt 2  Stomach Pain  Neck Pain  Young Hunt 3  Stomach Pain  Neck Pain | 20·58  23·96    20·03  30·46    42·28  37·98 | Prevalence, severity and risk factors of asthma, rhinitis and eczema in a large group of Chinese schoolchildren  Persistent cough in the past year | 10.4 |  |  |  |  |
| Young HUNT study  Young Hunt 1  Young Hunt 2  Young Hunt 3 | 29·67  36·77  29·78 |  |  |  |  |  |  |  |  |

* “frequent or severe headaches including migraine”

** “Frequent or chronic difficulties with repeated or chronic physical pain…including headaches…”

Table 2: Displays prevalence (%) of high-frequency physical symptoms (headache, general pain, cough, pulmonary symptoms, fatigue) in children and adolescents across studies, by symptom and age group where available.
