# Supplementary material for: Pre‐Pandemic Prevalence of Post COVID‐19 Condition Symptoms in Adolescents
Source: Acta Paediatr. 2025 Jun 6;114(9):2116–23. doi: 10.1111/apa.70123 (PMC12336932; doi:10.1111/apa.70123)
Supplement: Supplementary file 3 — Table S3. [file APA-114-2116-s003.docx]

**Supplementary Table 3: Prevalence by Study for Other Symptoms**

| **Study** | **Diarrhoea** | **Study** | **Constipation** | **Study** | **Problem swallowing** | **Study** | **Pulmonary Embolism** | **Study** | **Enuresis** |
| --- | --- | --- | --- | --- | --- | --- | --- | --- | --- |
| Young HUNT study  Young Hunt 1  Young Hunt 2  Young Hunt 3 | 5·54  5·99  1·24 | Young HUNT study  Young Hunt 1  Young Hunt 2  Young Hunt 3 | 7·19  7·45  1·37 | National Survey of Children’s Health (US population study)  2017-8  2019 | 1·5   (95% CI  [1.1 - 2.0])  1·1   (95% CI  [0.9 - 1.5]) | Young HUNT study  Young Hunt 1  Young Hunt 2 | 12·11  11·98 | PHDCN - Child Behaviour Checklist  Parent/carer reported symptoms  Wave 1, cohort 12  Wave 1, cohort 15 | 4·8  1·9 |

| **Study** | **Hallucinations** | **Study** | **Inflammatory Condition** | **Study** | **Learning difficulties** | **Study** | **Endocrine disorders** |
| --- | --- | --- | --- | --- | --- | --- | --- |
| Project on Human Development in the Chicago Neighbourhoods  PHDCN - Youth Self Report  Wave 1, cohort 12  Wave 1, cohort 15  PHDCN - Child Behaviour Checklist  Parent/carer reported symptoms  Wave 1, cohort 12  Wave 1, cohort 15 | 22·6 (Auditory)  18·9 (Visual)  12·4 (Auditory)  12·3 (Visual)  3·4 (Auditory)  2·7 (Visual)  2·6 (Auditory)  2·9 (Visual) | Finnish IEQ and Symptoms study  Grade 3-6 pupils  Grade 7-9 pupils | 8·5  6·1 | National Longitudinal Study of Youth | 7·5 | China Nutrition and Health Surveillance of Children and Lactating Women  10-14 years old  15-17 years old | 0·14  0·39 |
|  |  | National Survey of Children’s Health (US population study)  2016  2017-8 | 0·8  0·7 | National Survey of Children’s Health (US population study)  2016  2017-8  2019 | 8·5  (95% CI  [7.7 - 9.4])  8·4  (95% CI  [7.6 - 9.2])  8·6  (95% CI  [7.5 - 9.9]) | National Comorbidity Study-Adolescent Supplement | 0·87 |
|  |  |  |  |  |  | National Longitudinal Study of Youth | 0·2 |

| **Study** | **Chronic respiratory failure** | **Study** | **Anxiety** | **Study** | **Nervousness** | **Study** | **Psychiatric problem** |
| --- | --- | --- | --- | --- | --- | --- | --- |
| Children's Health Study  Bronchitic Symptoms Age 10 (4602)  Bronchitic Symptoms Age 15 | 44·03  33·43 | Mental health of children and young people Surveys (NHS digital) 2017  Boys  Girls  Boys  Girls  All 11-16  All 17-19 | 3·9  5·3  3·5  5·3  7·9  13·1 | Hawaiian High Schools Health Survey | 26·5 | Health Behaviours of School age Children  Total (all) | 24 |
| National Longitudinal Study of Youth | 8·2 | National Survey of Children’s Health (US population study)  2016  2017-8  2019 | 6·6  (95% CI  [5.7 - 7.6])  6·1  13·9  (95% CI  [12.5 - 15.3]) | Health Behaviours of School age Children  11 year old boys  11 year old girls  13 year old boys  13 year old girls  15 year old boys  15 year old girls  Total (all) | 33  37  39  57  54  65  44 | National Survey of Children’s Health (US population study)  2016  2017-8  2019 | 6·1  (95% CI  [5.5 - 6.8])  25·1  (95% CI  [23.9 - 26.4])  12·2  (95% CI  [11.1 - 13.4]) |
| National Survey of Children’s Health (US population study)  2016  2017-8  2019 | 10·4  9·8  10·9 | Project on Human Development in the Chicago Neighbourhoods  PHDCN - Youth Self Report  Wave 1, cohort 12  Wave 1, cohort 15  Wave 2, cohort 12  Wave 2, Cohort 15  Wave 3 cohort 9  Wave 3, cohort 12  PHDCN - Child Behaviour Checklist  Wave 1, cohort 12  PHSCN - Parent/carer reported symptoms  Wave 1, cohort 15  Wave 2, cohort 12  Wave 2, cohort 15  Wave 3, cohort 9  Wave 3, cohort 12 | 31·2  33·8  32·7  35·2  38·1  37·1  16·3  22·8  19·9  22·8  21·4  22·8 | National Comorbidity Study-Adolescent Supplement | 22·93 | Prevalence of mental health problems in schools | 18·5 |
|  |  |  |  | Project on Human Development in the Chicago Neighbourhoods  PHDCN – Youth Self Report  Wave 1, cohort 12  Wave 1, cohort 15  Wave 2, cohort 12  Wave 2, cohort 15  Wave 3 cohort 9  Wave 3, cohort 12  PHDCN – Parent/carer reported symptoms  wave 1, cohort 12  wave 1, cohort 15  Wave 2, cohort 12  Wave 2, cohort 15  Wave 3, cohort 9  Wave 3, cohort 12 | 48·6  57·5  50·9  54·9  50·8  54·9  29·4  35·5  31·9  36·8  26·7  35·2 | Time trends in adolescent mental health  Boys  Girls  Total | 16·7  (95% CI  1.44 [.9–2.3]])  13·1  (95% CI  1.59 [.95–2.7])  14·9  (95% CI  1.50 [1.1–2.0]) |
|  |  |  |  |  |  | Tokyo TEEN Cohort | 53·46 |

| **Study** | **Joint pain**  **/ Swelling** | **Study** | **Sadness** | **Study** | **Depression** | **Study** | **General wellbeing** |
| --- | --- | --- | --- | --- | --- | --- | --- |
| Avon Longitudinal Study of Children and Parents  Teenage focus  survey 2 | 9·5 | Avon Longitudinal Study of Children and Parents | 8·6  8·8 | Health Behaviours of School age Children  Total (girls)  Total (boys)  Total (all) | 46  31  22 | Children’s Wellbeing Measures ONS  2012  2013  2014  2015 | 84·2  84·2  84·3  85·3 |
| National Comorbidity Study-Adolescent Supplement  Arthritis | 2·21 | Hawaiian High Schools Health Survey | 34·7 | Mental health of children and young people Surveys (NHS digital) 2017  Boys  Girls  Boys  Girls  All 11-16  All 17-19 | 1·7  1·9  0·7  1·6  2·7  4·8 | Health Behaviours of School age Children  Total (girls)  Total (boys) | 66  55 |
| National Longitudinal Study of Adolescent Health | 6·4 | Health Behaviours of School age Children  11 year old boys  11 year old girls  13 year old boys  13 year old girls  15 year old boys  15 year old girls  Total (all) | 26  30  32  52  38  62  38 | National Comorbidity Study-Adolescent Supplement | 15·2 | Millenium cohort study - Age 14 sweep | 83·9 |
| National Survey of Children’s Health (US population study)  Arthritis  2016  2017-8 | 0·8  0·7 | Millenium cohort study - Age 14 sweep | 59·7 | National Longitudinal Study of Adolescent Health | 9 | National Comorbidity Study-Adolescent Supplement | 3·37 |
| Young HUNT study  Young Hunt 1  Young Hunt 2 | 16·68  16·83 | National Comorbidity Study-Adolescent Supplement | 11 | National Survey of Children’s Health (US population study)  2016  2017-8  2019 | 6·1  (95% CI  [5.5 - 6.8])  6·3  (95% CI  [5.7 - 6.9])  7·2  (95% CI  [6.3 - 8.3]) | National Longitudinal Study of Adolescent Health | 93·9 |
|  |  | National Longitudinal Study of Youth | 31·7 | Olympic Regeneration in East London (ORIEL)  Male  Female | 18·4  27·5  2.06 (95% CI  [1.60,2.65]) | National Longitudinal Study of Youth | 94·9 |
|  |  | Project on Human Development in the Chicago Neighbourhoods  PHDCN – Youth Self Report  Wave 1, cohort 12  Wave 1, cohort 15  Wave 2, cohort 12  Wave 2, cohort 15  Wave 3, cohort 9  Wave 3, cohort 12  PHDCN – Parent/carer reported symptoms  Wave 2, cohort 12  Wave 2, cohort 15  Wave 3, cohort 9  Wave 3, cohort 12 | 28·7  35·5  21·2  29·1  19·8  22·2  21·2  29·7  21·4  32·7 | Prevalence of mental health problems in schools | 18·4  2.93 (95% CI [2.73–3.14]) | National Survey of Children’s Health (US population study)  2016  2017-8  2019 | 87·6  (95% CI  [86.4 - 88.7])  87·2  (95% CI  [86.0 - 88.4])  87·1  (95% CI  [85.5 - 88.5]) |
|  |  | Understanding Society: Longitudinal Teaching Dataset,  Waves 1-9, 2009-2018 | 23·7 | Project on Human Development in the Chicago Neighbourhoods  PHDCN – Youth Self Report  Wave 1, cohort 12  Wave 1, cohort 15  Wave 2, cohort 12  Wave 2, cohort 15  Wave 3 cohort 9  Wave 3, cohort 12  PHDCN – Parent/carer reported symptoms  Wave 2, cohort 12  Wave 2, cohort 15  Wave 3, cohort 9  Wave 3, cohort 12 | 28·7  35·5  21·2  29·9  19·8  22·2  21·2  29·7  21·4  32·7 | Olympic Regeneration in East London (ORIEL)  Male  Female | 21·8  26·8  1.67 (95% CI  [1.32,2.12]) |
|  |  |  |  |  |  | Young HUNT study  Young Hunt 1  Young Hunt 2  Young Hunt 3 | 89·03  87·46  89·19 |
|  |  |  |  |  |  | Online social networking addiction and depression: The results from a large-scale prospective cohort study in Chinese adolescents | 24.6 |
|  |  |  |  |  |  | What about Youth Survey | 85 |

Supplementary Table 3: Displays prevalence (%) of less high prevalence symptoms across studies, including gastrointestinal, neurological, endocrine, and psychiatric symptoms.
