# Supplementary material for: Pre‐Pandemic Prevalence of Post COVID‐19 Condition Symptoms in Adolescents
Source: Acta Paediatr. 2025 Jun 6;114(9):2116–23. doi: 10.1111/apa.70123 (PMC12336932; doi:10.1111/apa.70123)
Supplement: Supplementary file 4 — Table S4. [file APA-114-2116-s002.docx]

**Supplementary Table 4: Symptom Frequency and Prevalence (%) with Median and IQR**

| **Symptom** | **Frequency of Symptom Prevalence** | **Prevalence (%)**  **Median (IQR)** |
| --- | --- | --- |
| Anxiety | 21 | 19·90 (6.60, 31.20) |
| Body weight changes | 1 | 56·80 |
| Chronic respiratory failure | 6 | 10·65 (9.95, 27.80) |
| Constipation | 3 | 7·19 (4.28, 7.32) |
| Cough | 30 | 13·60 (9.52, 32.36) |
| Depression | 29 | 21.20 (6.30, 27.50) |
| Diarrhoea | 3 | 5·54 (3.39, 5.77) |
| Endocrine disorders | 4 | 0·30 (0.19, 0.51) |
| Enuresis | 2 | 3·35 (2.63, 4.08) |
| Fatigue | 9 | 20.50 (14.89, 29.00) |
| General wellbeing | 19 | 85.3 (84.05, 88.32) |
| Hallucinations | 8 | 7·85 (2.85, 14.03) |
| Headache | 33 | 30.00 (26.82, 56.40) |
| Infectious symptoms | 2 | 18·10 (16.05, 20.15) |
| Inflammatory Condition | 4 | 3·45 (0.78, 6.70) |
| Joint Pain or swelling | 7 | 7·95 (1.86, 12.87) |
| Learning difficulties | 4 | 8·45 (8.18, 8.53) |
| Nervousness | 20 | 41.50 (34.65, 54.23) |
| Pain | 37 | 20.00 (14, 32.50) |
| Problems Swallowing | 2 | 1·30 (1.20, 1.40) |
| Psychiatric problem | 6 | 15·80 (13.55, 18.05) |
| Pulmonary symptoms | 11 | 11·60 (9.00, 19.15) |
| Sadness | 20 | 29·40 (21.6, 34.80) |

Supplementary Table 4: Displays the frequency, prevalence (%) with Median and Interquartile Range for each symptom extracted from the papers. Symptoms without associated prevalence data have been excluded from the table.
