# Supplementary material for: Pre‐Pandemic Prevalence of Post COVID‐19 Condition Symptoms in Adolescents
Source: Acta Paediatr. 2025 Jun 6;114(9):2116–23. doi: 10.1111/apa.70123 (PMC12336932; doi:10.1111/apa.70123)
Supplement: Supplementary file 7 — Table S7. [file APA-114-2116-s005.docx]

**Table S7: Prevalence of Symptoms Across Studies: A Comparative Summary (continued)**

| **Paper/Symptom** | Nervousness | Pain | Problems Swallowing | Psychiatric Problems | Pulmonary Symptoms | Sadness |
| --- | --- | --- | --- | --- | --- | --- |
| Avon Longitudinal Study of Children and Parents |  | x |  |  | x |  |
| Children's Health Study |  |  |  |  | x |  |
| Childrens Wellbeing Measures ONS |  |  |  |  |  |  |
| China Nutrition and Health Surveillance of Children and Lactating Women |  |  |  |  |  |  |
| Hawaiian High Schools Health Survey | x |  |  |  |  | x |
| Health Behaviours of School age Children | x | x |  | x |  | x |
| Finnish IEQ and Symptoms study |  |  |  |  | x |  |
| Leicestershire Respiratory Cohort |  |  |  |  |  |  |
| Mental health of children and young people Surveys (NHS digital) 2017 |  |  |  |  |  |  |
| Millenium cohort study - Age 14 sweep |  |  |  |  |  | x |
| National Comorbidity Study-Adolescent Supplement | x | x |  |  |  |  |
| National Longitudinal Study of Adolescent Health |  | x |  |  | x |  |
| National Longitudinal Study of Youth |  |  |  |  |  | x |
| Olympic Regeneration in East London (ORIEL) |  |  |  |  |  |  |
| Online social networking addiction and depression |  |  |  |  |  |  |
| Prevalence of mental health problems in schools |  |  |  | x |  |  |
| Prevalence, severity and risk factors of asthma, rhinitis and eczema in a large group of Chinese schoolchildren |  |  |  |  |  |  |
| Project on Human Development in the Chicago Neighbourhoods | x | x |  |  |  | x |
| Time trends in adolescent mental health |  |  |  | x |  |  |
| Tokyo TEEN Cohort |  |  |  | x |  |  |
| Tracking Adolescents Individual Lives Surveys |  | x |  |  |  |  |
| National Survey of Children’s Health |  | x | x | x | x |  |
| What about Youth Survey |  |  |  |  |  |  |
| Young HUNT study |  | x |  |  | x |  |
| Understanding Society |  |  |  |  |  | x |
| **Total** | **4** | **8** | **1** | **5** | **6** | **6** |

Table S7: Displays the frequency of each symptom extracted from the papers. Symptoms without associated prevalence data have been excluded from the table.
